# Comparison of approaches for source attribution of ESBL-producing *Escherichia coli* in Germany

**DOI:** 10.1371/journal.pone.0271317

**Published:** 2022-07-15

**Authors:** Sara Perestrelo, Guido Correia Carreira, Lars Valentin, Jennie Fischer, Yvonne Pfeifer, Guido Werner, Judith Schmiedel, Linda Falgenhauer, Can Imirzalioglu, Trinad Chakraborty, Annemarie Käsbohrer

**Affiliations:** 1 Biological Safety, German Federal Institute for Risk Assessment, Berlin, Germany; 2 Nosocomial Pathogens and Antibiotic Resistance, Robert Koch Institute, Wernigerode, Germany; 3 Institute of Medical Microbiology, Justus Liebig University, Giessen, Germany; 4 Institute of Hygiene and Environmental Medicine, Justus Liebig University, Giessen, Germany; 5 German Center for Infection Research (DZIF), Partner Site Giessen-Marburg-Langen, Campus Giessen, Giessen, Germany; 6 Hessisches universitäres Kompetenzzentrum Krankenhaushygiene (HuKKH), Giessen, Germany; 7 Veterinary Public Health and Epidemiology, University of Veterinary Medicine, Vienna, Austria; Amphia Ziekenhuis, NETHERLANDS

## Abstract

Extended-spectrum beta-lactamase (ESBL)-producing *Escherichia (E*.*) coli* have been widely described as the cause of treatment failures in humans around the world. The origin of human infections with these microorganisms is discussed controversially and in most cases hard to identify. Since they pose a relevant risk to human health, it becomes crucial to understand their sources and the transmission pathways. In this study, we analyzed data from different studies in Germany and grouped ESBL-producing *E*. *coli* from different sources and human cases into subtypes based on their phenotypic and genotypic characteristics (ESBL-genotype, *E*. *coli* phylogenetic group and phenotypic antimicrobial resistance pattern). Then, a source attribution model was developed in order to attribute the human cases to the considered sources. The sources were from different animal species (cattle, pig, chicken, dog and horse) and also from patients with nosocomial infections. The human isolates were gathered from community cases which showed to be colonized with ESBL-producing *E*. *coli*. We used the attribution model first with only the animal sources (Approach A) and then additionally with the nosocomial infections (Approach B). We observed that all sources contributed to the human cases, nevertheless, isolates from nosocomial infections were more related to those from human cases than any of the other sources. We identified subtypes that were only detected in the considered animal species and others that were observed only in the human population. Some subtypes from the human cases could not be allocated to any of the sources from this study and were attributed to an unknown source. Our study emphasizes the importance of human-to-human transmission of ESBL-producing *E*. *coli* and the different role that pets, livestock and healthcare facilities may play in the transmission of these resistant bacteria. The developed source attribution model can be further used to monitor future trends. A One Health approach is necessary to develop source attribution models further to integrate also wildlife, environmental as well as food sources in addition to human and animal data.

## Introduction

Extended-spectrum beta-lactamase (ESBL)-producing *Escherichia* (*E*.) *coli* were first mentioned in 1983 [1] and have been widely known for several years as the cause of treatment failures around the world. ESBLs are enzymes hampering the use of beta-lactam antibiotics such as penicillins and early cephalosporins, but also cephalosporins of the 3^rd^ and 4^th^ generation and monobactams. This broad activity is the reason, why they are designated as beta-lactamases with an extended spectrum. Given that, they pose an additional risk for patients suffering from a serious infection of ESBL-producing *E*. *coli* as treatment may be compromised. The ESBL encoding genes may be further transmitted to other bacterial clones, or species through horizontal gene transfer due to their location on mobile genetic elements (MGE) such as plasmids [2]. Apart from this, *E*. *coli* harboring ESBLs are frequently multidrug-resistant (MDR), to not only β-lactams but also to other antimicrobial classes, such as quinolones, aminoglycosides, tetracyclines and sulfonamides [3], with responsible genes being co-located on the same MGE as the ESBL genes or other MDR plasmids in the same host, or in the hosts chromosome.

The origin of colonization with ESBL-producing *E*. *coli* amongst humans is controversial and the role of the environment, foodstuffs, pets, wildlife, livestock animals and healthcare facilities is still under discussion. Furthermore, the routes and direction of transmission between animals and humans are not clear and it is more likely to be rather bilateral than unilateral, meaning that humans may also contribute to the increased presence of ESBL-producing bacteria amongst animals and the environment. In addition, the human-to-human transmission has been further mentioned with more studies referring to the increasing role of travelers, clinical patients and farmers as risky vehicles of ESBL-positive bacteria into the general community [4, 5].

In Germany, data on the prevalence of ESBL-producing *E*. *coli* was gathered in 2011–2013 by the RESET-consortium (http://www.reset-verbund.de), which has broadened up the understanding of the current situation in the country. Many studies have described and characterized the presence of ESBL-carrying *E*.*coli* in Germany in different environments and hosts; on animal farms [6, 7], in the environment [8], in hospitals [9] and in the general human population [10].

Source attribution modeling based on microbial subtyping has been used to identify potential animal and non-animal sources of different zoonotic bacteria in various countries, such as Denmark (for *Salmonella*) [11], Netherlands (for Shiga toxin-producing *E*. *coli* (STEC)) [12] and New Zealand (for Campylobacter) [13]. These mathematical models use subtyping data from molecular as well as phenotypical techniques.

In 2019, Jabin et al. [14] used two different source attribution models to predict the origin of *Salmonella* amongst humans from different food sources (broilers, laying hens, pigs and turkeys) in Germany. Nevertheless, there was still lacking a similar study in Germany regarding ESBL-producing *E*. *coli*. In this study, we aim to analyze the available data from the RESET project in Germany and to apply a Bayesian source attribution model to gain more knowledge about the origin of ESBL-producing *E*. *coli* cases in the general population. The sources considered in this study are cattle, chicken, pigs, horses, dogs and nosocomial cases.

This kind of analysis can add useful information for the important public health question of at which point(s) interventions against the rise of antibiotic resistance may have the largest effect. If the largest part of resistant bacteria in humans are attributable to livestock (and to the corresponding foods), this might suggest that focusing on animals in order to reduce resistances might be the most effective thing to do. This could include studying mitigating strategies against the spread of resistant bacteria in livestock like for example veterinarian antimicrobial (also called antibiotic) stewardship [15–17] or husbandry practices [18, 19]. If, on the other hand, the largest part of resistances circulates within human populations then this might suggest that it is more effective focusing on the human side in order to mitigate the rise of resistant strains of bacteria (again for example through antibiotic stewardship [20]). If finally both, human and animal sources play a considerable role a broader one health approach seems more appropriate [21, 22].

## Material and methods

Basis for this analysis were *E*. *coli* isolates from several studies on the prevalence and phenotypic and genotypic characteristics of ESBL-producing *E*. *coli* in Germany conducted firstly within the research consortium RESET and then recovered for the present study within the DiSCoVer project (One Health EJP consortium).

The available data is from healthy livestock animals (broilers, fattened pigs, dairy cattle and beef cattle), horses and dogs from Germany. Clinical isolates from livestock animals were excluded, nevertheless, all isolates from pets (dogs and horses) originated from clinical samples. As regards to humans, the focus was set on isolates from the healthy community and patients with nosocomial infections.

Based on phenotypic and genotypic characteristics, the isolated *E*. *coli* isolates were grouped into “subtypes”. Different approaches were used here in order to study how source attribution results change with the characteristics considered for defining the subtypes. In the following, we will use the terms *type*, *subtype* and *ESBL-type* interchangeably.

### Origin of non-human isolates

Three studies collected faecal, dust and environmental samples from farms with healthy livestock animals. In the first study, seven conventional broiler and seven pig-fattening farms from different regions in Germany were sampled. Each farm was visited at the beginning, middle and end of the fattening period [7, 23, 24]. To avoid overrepresentation of isolates with specific characteristics which might occur through repeated sampling of the same group of birds, only one isolate of each unique type (as defined above) collected within the three visits of the same farm were included to simulate a cross-sectional study.

The other two studies were cross-sectional studies. One investigated 34 broiler, 48 fattening pig and 42 cattle farms in several regions of Germany, except Bavaria [25, 26] and the other 30 mixed dairy and beef cattle farms and 15 beef cattle farms in Bavaria [27].

All livestock samples were tested for the presence of ESBL-producing *E*. *coli* using selective MacConkey agar containing 1 mg/L cefotaxime [23] by study partners. Isolates from positive samples were sent to the National Reference Laboratory for Antimicrobial Resistance at the Federal Institute for Risk Assessment (BfR) in Berlin, Germany for confirmation and further molecular characterization [28, 29].

Regarding the dog and horse *E*. *coli* isolates, these originated from a collection of 600 *Enterobacteriaceae* strains from the Institute of Hygiene and Infectious Diseases of Animals (IHIT) in Giessen that were selected on MacConkey agar containing 1 mg/L cefotaxime [30]. The isolates were PCR screened for the presence of ESBL genes and genes contributing to (fluoro)quinolone resistance by the Institute of Medical Microbiology in Giessen. In the present study, we selected them based on the presence of ESBL genes [30].

### Origin of human isolates

Isolates from one study of the general population and one study on nosocomial infections were included. In the first study, faeces from the healthy Bavarian community showing no symptoms of gastroenteritis were inoculated on MacConkey agar supplemented with 1 mg/L cefotaxime. Positive samples were considered as “community associated cases” colonized with ESBL-producing *E*. *coli* [10] and in the following we will refer to them as *cases* for short.

In the second study, a laboratory network operating throughout Germany collected ESBL-producing *E*. *coli* isolates from nosocomial infections (isolated in hospitals at least 48 hours after submission) on the basis of their phenotypic resistance to 3rd generation cephalosporins as identified by commercial selective media [9]. The isolates were then forwarded for further characterization to the National Reference Centre at the Robert Koch Institute (RKI) in Wernigerode, Germany [31, 32]. The nosocomial isolates included in our study were both tested for cefotaxime and ceftadizime. As we only used archived data, which was fully anonymous and did not contain any information relating their participants, no ethics committee was consulted in the present study.

### Phenotypic and genotypic isolate characterization

All ESBL-producing *E*. *coli* were further analyzed in order to determine phenotypic and genotypic information related to their resistance-associated traits.

A large panel of antibiotics (amikacin, ampicillin, ceftazidime, cefotaxime, cefepime, chloramphenicol, ciprofloxacin, gentamicin, ertapenem and imipenem) was tested using minimum inhibitory concentration (MIC) or disk diffusion (DD) and evaluated according to EUCAST clinical breakpoints (www.eucast.org). For the present analysis, intermediate results were subsequently categorized as resistant.

In the present modelling approach, four different antibiotics were considered: gentamicin (S≤2mg/L | S≥17mm with 10μg disc; aminoglycoside), ertapenem (S≤0.5mg/L | DD not used; carbapenem), ciprofloxacin (S≤0.5mg/L | S≥22mm with 5μg disc; fluoroquinolone) and chloramphenicol (S≤8mg/L | S≥17mm with 30μg disc; phenicol). The selection of the antibiotics was a compromise of the test panels used in the routine antibiotic susceptibility testing of the involved institutions. In addition, this selection was made to assure a heterogeneous antibiotic resistance pattern.

The focus of this study was solely on ESBL genes (*bla*_CTX-M_, *bla*_SHV_, *bla*_TEM_), therefore, all the subtypes linked to AmpC β-lactamases were excluded from our dataset. ESBL genes were detected by PCR and when necessary confirmed by sequence analysis of the PCR product (as for *bla*_SHV_, *bla*_TEM_) [29]. Usually, the *bla*_TEM_ gene was not sequenced if an ESBL gene (e.g. *bla*_CTX-M_ and *bla*_SHV_) was detected in the same isolate; in this case the *bla*_TEM_ gene is denominated as *neg* (as in “negative”). If the *bla*_TEM_ gene was sequenced, either the gene denomination is given (e.g. *bla*_TEM-52_) or we denominated it as neg in case *bla*_TEM_ was not identified. Furthermore, a PCR assay was performed to differentiate *E*. *coli* isolates into the four major phylogenetic groups (A, B1, B2, D) [28, 33].

Only isolates of an ESBL-type with a complete typing dataset were considered in this modelling approach. Table 1 summarizes the final number of ESBL-producing *E*. *coli* isolates per group and study.

**Table 1:** Number of ESBL-producing *E*. *coli* isolates (n = 935) used in the modelling approaches.

Source		No. isolates
Broiler		53
Cattle		183
Pig		164
Horse		81
Dog		29
Human	Nosocomial infection	212
	General Population	213
Total		935

### The model

The model used in this approach is a variation [34] of the well-established Bayesian source attribution model originally developed for *Salmonella* [11, 35]. It compares the subtype distribution of a bacterial species in animals with the subtype distribution in humans achieved by the same subtyping methods. The model calculates the number of human cases that can be attributed to each of the potential sources (animal species and nosocomial infections) based on the assumption that there is a pathway from these sources into the general population.

The aim of the present modelling approach was to use microbial and molecular information on ESBL-producing *E*. *coli* to estimate the contribution of different potential sources to colonize or infect humans. The following equations were used to define the model:

$$o_{i}∼Poisson\;\left(\sum_{j}\lambda_{ij}\right)$$ (1)

$$\lambda_{ij}=M_{j}p_{ij}q_{i}a_{j}$$ (2)

$$\begin{array}{c} a_{j}∼uniform(0,1000)\\ q_{i}∼uniform(0,1000) \end{array}$$ (3)

where *a*_*j*_ and *q*_*i*_ are prior distributions.

The parameters used to feed the model are explained in Table 2 and in line with those already previously described by Hald and co-workers [11].

**Table 2:** Parameters used in the model.

Parameters	Description				
*i*	index of subtype				
*j*	index of source				
*o*_*i*_	observed human cases of subtype *i*				
*p*_*ij*_	observed prevalence of subtype *i* in source *j*				
*λ*_*ij*_	expected number of human cases of subtype *i* in source *j*				
*a*_*j*_	unknown source-dependent parameter of source *j*				
*q*_*i*_	unknown subtype-dependent parameter of subtype *i*				
*M*_*j*_	consumption of source *j*				

The amount of food consumed (*M*_*j*_*)* and the prevalence (*p*_*ij*_) had been introduced into the model by Hald et al. [11] to weight the model results. Due to the interest to use pets and humans as a potential source, the observation of high prevalence rates in all animal species and the expected low impact of the amount of food consumed, it was decided to use fixed values for these parameters (*M*_*j*_ = 1 and *p*_*ij*_ = 1).

The model was implemented by using the software R (version 4.0.3) [36] and JAGS which uses Markov Chain Monte Carlo methods to sample from the posterior distribution. In order to use JAGS with R we used the R package *rjags* [37]. Five Markov chains were used. JAGS features a process called adaptation (also called tuning). In this process JAGS tunes internal parameters of the Gibbs sampler in order to optimize its efficiency for sampling the posterior distribution. Users can control the extension of this tuning process via the *n*.*adapt* parameter. We set the *n*.*adapt* parameter to 1000. After that, we ran a burn-in period of 400 iterations followed by 500 sampling iterations. We applied thinning, which in our case means that of the 500 sampling values only every tenth was kept for each chain. The R code is available upon request.

### Defining subtypes and excluding data

We generated three different subtype sets of the same set of isolates, which vary by the incorporation of the information: i) (Set 1) the ESBL gene pattern [*bla*_CTX-M_ | *bla*_SHV_ | *bla*_TEM_], ii) (Set 2) additionally to Set 1 the phylogenetic groups [A, B1, B2 or D] and iii) (Set 3) additionally to Set 2 the antibiotic resistance pattern (**ARP**) of four selected antibiotics [gentamicin, ertapenem, ciprofloxacin, and chloramphenicol] with ‘R’ coding for resistant and ‘S’ for susceptible.

The character string **1.neg.1.D.RRSR** is an example for describing subtypes in Set 3. In the first position (positions are separated by a dot), “1” stands for the presence of *bla*_CTX-M-1_, “neg” in the second position stands for the absence of *bla*_SHV_ and “1” in the third position stands for the presence of *bla*_TEM-1_. Since *bla*_TEM-1_ and *bla*_TEM-135_ don´t confer resistance to 3rd generation cephalosporins (non-ESBL), the subtypes only containing these *bla*_TEM_ genes (e.g. neg.neg.1) were excluded from the data that went into the model calculations. In the fourth position, “D” reflects the phylogenetic group and in the last position, the resistance pattern consisting of resistance (R) to gentamicin, ertapenem, chloramphenicol and sensitivity (S) to ciprofloxacin is depicted.

The model requires that for each single bacterial type at least one human case. In a first step, the model looks whether at the same time at least one single bacterial type was isolated in one of the sources considered. We will call these types from now on *matching types* because human cases can in principle be matched to potential sources.

Consequently, there were two kinds of bacterial types in which this matching is not possible and had therefore to be excluded from the data used to run the model. First, bacterial types never found in a human case had to be excluded before running the model. For descriptive reasons, we denominate these types as *other* since they cannot explain any human cases. The second kind of type we excluded for running the model are types only found in human cases but never in a source. This means that we have no clue from which source these types originated. Therefore, we use the term *unknown* alone or in concert with “types” or “sources”, which is a shorthand for “types or isolates associated to unknown sources”.

We did not include other and unknown types in the data used to run the model, since the model cannot deal with them as they make it impossible to associate human cases to possible sources. Nevertheless, after running the model to complete the source attribution process, we manually included the number of cases related to unknown sources in our analysis, since these give an important information about the magnitude of attributed (*matched*) cases amongst the different sets. This means that the number of human cases allocated to unknown sources were not an outcome of the mathematical model, but were instead integrated on a later stage in the data analysis.

### Modelling scenarios

Two different scenarios attributing isolates from several sources to isolates from healthy humans colonized with ESBL-producing *E*. *coli* were analyzed. The first scenario (Approach **A**) considers only the potential animal sources broilers, cattle, fattening pig, horses and dogs. The second scenario (Approach **B**) additionally includes as potential source humans infected with hospital acquired ESBL-producing *E*. *coli*. The latter approach stands for the potential spread of bacteria from hospitals (nosocomial infections) into the community.

### Modifications of considered antibiotics (modified Approach B)

The third scenario reflects modifications of including each antibiotic on the model. The Approach B was modelled only using three antibiotics instead of four in order to check if there were major changes on the allocations when one of the antibiotics was absent. For this scenario, only Set B3 was used. All four antibiotic combinations, each with one of the considered antibiotics removed, were used in this scenario.

## Results

### Approaches considering animal sources

#### Set A1

In Approach A, overall 723 isolates were included. Set A1 incorporated only information on the presence of the ESBL genes *bla*_CTX-M_, *bla*_SHV_ and *bla*_TEM_, which led to the definition of 28 different subtypes. Among these, 11 were categorized as other types (with 38 isolates) and 6 were unknown types (with 22 isolates). The remaining 663 isolates allocated to 11 matching types (Table 3) were used for running the model.

**Table 3:** Number of matching, unknown and other types for Set A1 and B1.

	Sources							
	Cases (B)	Nosocomial	Cases (A)	Cattle	Chicken	Dog	Horse	Pig
**Proportions of isolates with certain types (in %)**								
15.neg.1	26,3	17,2	26,3	2,7	0	17,9	31,8	0
15.neg.neg	18,8	36,4	18,8	21,3	7	39,3	13,6	7,9
1.neg.neg	17,8	18,7	17,8	55,7	41,9	10,7	21,2	71,7
14.neg.neg	8,9	4,5	8,9	8,7	0	0	0	3,3
1.neg.1	6,6	15,2	6,6	5,5	2,3	28,6	25,8	10,5
14.neg.1	5,6	1,5	5,6	1,1	0	3,6	0	0
neg.neg.52	2,3	1	2,3	0	30,2	0	0	3,9
neg.12.neg	1,4	0,5	1,4	0	11,6	0	0	0,7
neg.12.1	0,9	0	0,9	0,5	7	0	0	1,3
9.neg.1	0,5	0	0,5	0	0	0	1,5	0,7
2.neg.neg	0,5	1	0,5	4,4	0	0	6,1	0
55.neg.1	0,5	0,5	NA	0	0	0	0	0
32.neg.neg	0,5	0,5	NA	0	0	0	0	0
3.neg.neg	2,3	1,5	NA	0	0	0	0	0
27.neg.neg	3,8	1,5	NA	0	0	0	0	0
Types from unknown sources	3,3	0	10,3	0	0	0	0	0
**Absolute number of isolates for various type categories**								
Matching + unknown types	**213**	**198**	**213**	**183**	**43**	**28**	**66**	**152**
Matching types	206	198	191	183	43	28	66	152
Types from unknown sources	7	0	22	0	0	0	0	0
Other types	0	14	0	0	10	1	15	12
All types	213	212	213	183	53	29	81	164

The heat map shows the proportions of isolates with certain types among the sum of isolates with matching and unknown types for each source and each group of cases. Classification is according to scheme **Set 1** (resistance genes: “CTX-M.SHV.TEM”), for **Approach A** (only animal sources) and **Approach B** (with nosocomial isolates). The unknown reflects the isolates from the human population that had subtypes that could not be found in any of the considered source populations. The matching types displays the number of isolates per source that matched with human cases. The other type represents isolates with subtypes in the sources that were not found in the human cases. *All types* is the number of all isolates considered in this analysis. NA = not applicable.

**Fig 1:**
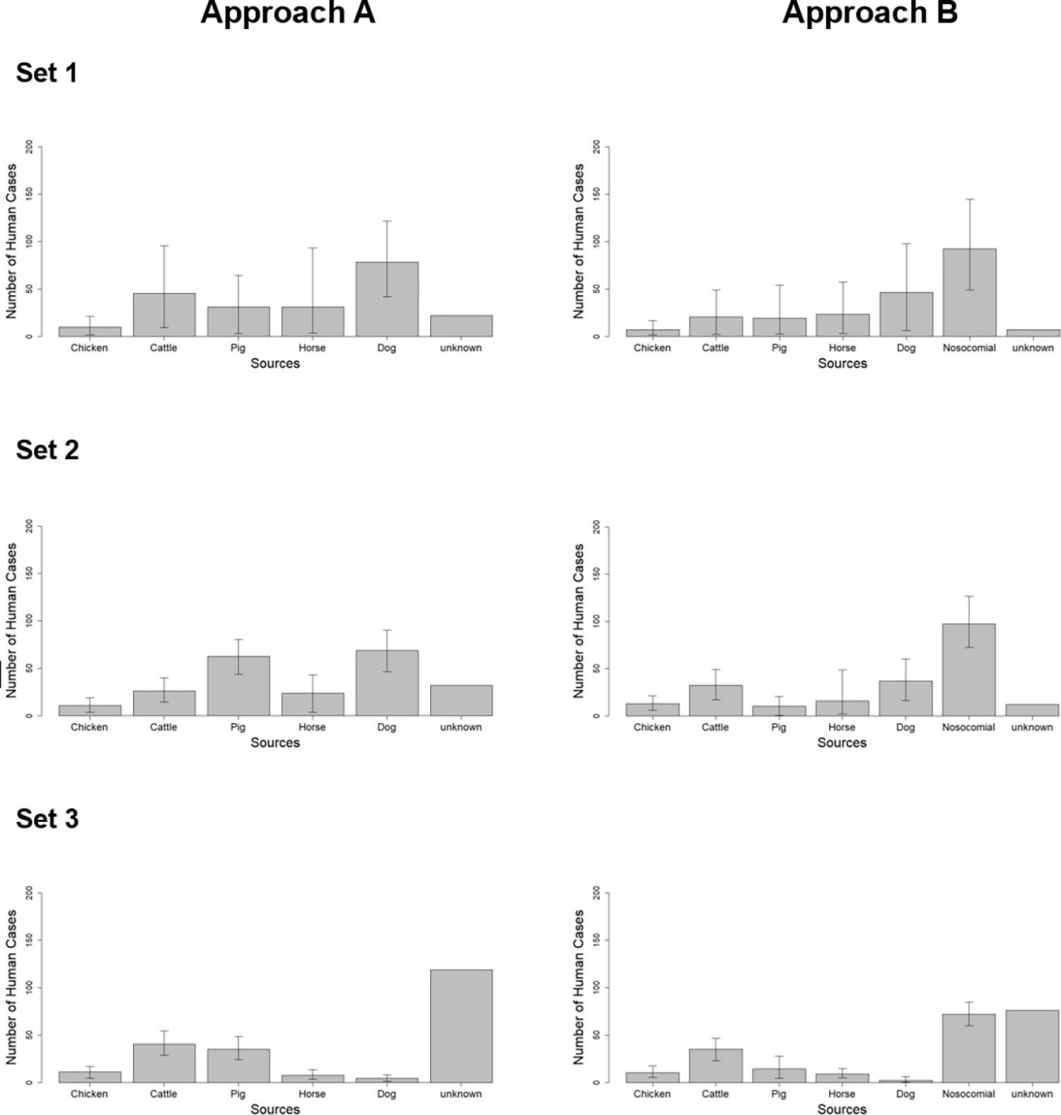
Source attribution modeling of ESBL-*E*. *coli* in human cases. Approach A–sources considered are chicken, cattle, pig, horse and dog. Approach B–sources considered are chicken, cattle, pig, horse, dog and hospitalized patients (nosocomial infections). Set 1, considers only ESBL-types; Set 2, considers ESBL-types and phylogroup of *E*. *coli*; Set 3, considers ESBL-types, phylogroup of *E*. *coli* and resistance pattern of four antimicrobials.

After running the model, the majority of the community cases were attributed to the sources dog (35.0%) and cattle (24.3%). Pig was attributed to 13.1% of the cases followed by horse (12.1%). Broiler accounted for only 5.1% of the human cases. The remaining 10.3% of human cases could not be attributed to any of the considered sources (see Set 1 in Approach A of Fig 1 and S1 Table).

#### Set A2

Adding to the gene type (Set A1) the information about the *E*. *coli* phylogenetic group (A, B1, B2 or D), the Set A2 comprised 64 different subtypes. From these subtypes, 12 were unknown types (with 32 isolates) and 24 were considered other types (with 48 isolates). The remaining 667 isolates, which belonged to 28 matching types (Table 4), were used to run the model.

**Table 4:** Number of matching, unknown and other types for Set A2 and B2.

	Sources							
	Cases (B)	Nosocomial	Cases (A)	Cattle	Chicken	Dog	Horse	Pig
**Proportions of isolates with certain types (in %)**								
15.neg.1.A	12,7	1,6	12,7	0	0	3,6	1,6	0
15.neg.neg.A	9,9	6,2	9,9	16,1	4,8	0	1,6	6,7
15.neg.1.D	9,9	5,2	9,9	0	0	3,6	11,3	0
1.neg.neg.A	8,9	3,6	8,9	16,7	14,3	0	6,5	41,3
14.neg.neg.D	6,1	1,6	6,1	0	0	0	0	0,7
1.neg.neg.D	5,6	2,6	5,6	12,2	7,1	3,6	0	4
15.neg.neg.D	4,7	6,8	4,7	1,7	0	7,1	1,6	0
14.neg.1.D	3,8	1	3,8	0	0	3,6	0	0
15.neg.neg.B2	2,8	18,2	2,8	0	0	3,6	0	0
1.neg.1.A	2,3	2,6	2,3	2,2	2,4	0	1,6	6,7
14.neg.1.A	1,9	0	1,9	1,1	0	0	0	0
1.neg.neg.B1	1,9	8,9	1,9	27,2	14,3	7,1	16,1	26
1.neg.1.D	1,9	2,6	1,9	2,2	0	10,7	3,2	0,7
neg.neg.52.A	1,4	0	1,4	0	19	0	0	1,3
15.neg.neg.B1	1,4	6,2	1,4	3,9	2,4	28,6	11,3	1,3
14.neg.neg.A	1,4	0	1,4	7,8	0	0	0	2,7
1.neg.neg.B2	1,4	4,2	1,4	0,6	7,1	0	0	1,3
1.neg.1.B1	1,4	6,2	1,4	0,6	0	17,9	19,4	3,3
neg.12.neg.B1	0,9	0	0,9	0	2,4	0	0	0
1.neg.1.B2	0,9	4,2	0,9	0,6	0	0	3,2	0
neg.neg.52.D	0,5	0,5	0,5	0	9,5	0	0	0
neg.neg.52.B1	0,5	0,5	0,5	0	2,4	0	0	2,7
neg.12.neg.D	0,5	0	0,5	0	7,1	0	0	0,7
neg.12.1.D	0,5	0	0,5	0	4,8	0	0	0,7
neg.12.1.A	0,5	0	0,5	0	2,4	0	0	0
2.neg.neg.A	0,5	0,5	0,5	3,3	0	0	1,6	0
15.neg.1.B1	0,5	3,1	0,5	2,8	0	10,7	21	0
14.neg.neg.B1	0,5	0,5	0,5	1,1	0	0	0	0
3.neg.neg.D	1,4	1	NA	0	0	0	0	0
27.neg.neg.B2	3,8	1,6	NA	0	0	0	0	0
15.neg.1.B2	3,3	7,8	NA	0	0	0	0	0
14.neg.neg.B2	0,9	2,6	NA	0	0	0	0	0
Types from unknown sources	5,6	0	15	0	0	0	0	0
**Absolute number of isolates for various type categories**								
Matching + unknown types	**213**	**192**	**213**	**180**	**42**	**28**	**62**	**150**
Matching types	201	192	181	180	42	28	62	150
Types from unknown sources	12	0	32	0	0	0	0	0
Other types	0	20	0	3	11	1	19	14
All types	213	212	213	183	53	29	81	164

The heat map shows the proportions of isolates with certain types among the sum of isolates with matching and unknown types for each source and each group of cases. Classification is according to scheme Set 2 (resistance genes: “CTX-M.SHV.TEM.Phylogroup”), for Approach A (only animal sources) and Approach B (with nosocomial infections). NA = not applicable. *See description Table 3.*

Using the ESBL gene and phylogenetic group information in the model (Set A2), dogs and pigs explained the majority of the human cases. Nevertheless, when compared to Set A1, the attributed proportion of human cases to the source dog (30.0% vs. 35%) and cattle (11.3% vs. 24.3%) decreased. In contrast, the attribution to the source pig (28.6% vs. 13.1%) increased considerably compared to Set A1. The attributed cases to chicken (3.8%) and to horse (11.3%) were quite similar to the previous model (Set A1). The number of cases attributed to an unknown source (15.0% vs. 10.3%) increased compared to Set A1 (see Set 2 of Approach A in Fig 1 and S1 Table).

#### Set A3

Considering additionally the antibiotic resistance pattern (ARP), Set A3 contained 186 different subtypes. Among these, 62 were unknown types (with 119 isolates) and 87 were other types (with 244 isolates). The remaining 360 isolates, which consisted of 37 matching types (Table 5), were used to run the model.

**Table 5:** Number of matching, unknown and other types for Set A3 and B3.

	Sources							
	Cases (B)	Nosocomial	Cases (A)	Cattle	Chicken	Dog	Horse	Pig
**Proportions of isolates with certain types (in %)**								
1.neg.neg.A.SSSS	6,6	3,4	6,6	16	11,8	0	0	40,9
1.neg.neg.D.SSSS	4,2	1,1	4,2	10,6	8,8	0	0	5,2
15.neg.neg.A.SSSS	2,3	0	2,3	1,1	0	0	0	0
15.neg.neg.A.SRSS	2,3	1,1	2,3	7,4	0	0	0	5,2
14.neg.neg.D.SSSS	2,3	1,1	2,3	0	0	0	0	0,9
1.neg.1.A.SSSS	2,3	2,3	2,3	2,1	2,9	0	0	4,3
15.neg.neg.D.SRSR	1,4	2,3	1,4	1,1	0	0	0	0
15.neg.neg.B1.SSSS	1,4	0	1,4	2,1	0	0	6,2	0
15.neg.neg.A.SRSR	1,4	4,6	1,4	0	0	0	0	0,9
1.neg.neg.B2.SSSS	1,4	3,4	1,4	1,1	8,8	0	0	0,9
1.neg.neg.B1.SSSS	1,4	6,9	1,4	24,5	17,6	0	6,2	23,5
1.neg.neg.A.SRSS	1,4	1,1	1,4	0	5,9	0	0	2,6
neg.neg.52.A.SSSS	0,9	0	0,9	0	23,5	0	0	0
15.neg.neg.D.SSSS	0,9	1,1	0,9	1,1	0	0	0	0
15.neg.neg.A.RSSS	0,9	0	0,9	0	0	0	6,2	0
14.neg.neg.A.SSSS	0,9	0	0,9	1,1	0	0	0	0
14.neg.1.A.RRSR	0,9	0	0,9	2,1	0	0	0	0
1.neg.neg.D.RSSS	0,9	1,1	0,9	2,1	0	14,3	0	0
1.neg.1.D.SSSS	0,9	2,3	0,9	1,1	0	0	0	0,9
1.neg.1.B1.RSSR	0,9	0	0,9	0	0	0	25	0
neg.neg.52.B1.SSSS	0,5	0	0,5	0	2,9	0	0	3,5
neg.12.neg.D.SSSS	0,5	0	0,5	0	5,9	0	0	0,9
neg.12.neg.B1.SSSS	0,5	0	0,5	0	2,9	0	0	0
neg.12.1.D.SRSS	0,5	0	0,5	0	2,9	0	0	0
15.neg.neg.D.RRSS	0,5	2,3	0,5	1,1	0	14,3	0	0
15.neg.neg.A.RSSR	0,5	0	0,5	1,1	0	0	0	0
15.neg.neg.A.RRSR	0,5	3,4	0,5	16	5,9	0	0	2,6
15.neg.1.D.RRSS	0,5	2,3	0,5	0	0	14,3	0	0
15.neg.1.A.RRSS	0,5	0	0,5	0	0	0	6,2	0
15.neg.1.A.RRSR	0,5	1,1	0,5	0	0	14,3	0	0
14.neg.neg.B1.SSSS	0,5	1,1	0,5	1,1	0	0	0	0
14.neg.neg.A.RRSS	0,5	0	0,5	0	0	0	0	0,9
1.neg.neg.B1.RSSR	0,5	0	0,5	1,1	0	0	37,5	0
1.neg.neg.A.SSSR	0,5	0	0,5	1,1	0	0	0	1,7
1.neg.neg.A.RSSR	0,5	0	0,5	3,2	0	0	12,5	2,6
1.neg.1.D.RRSR	0,5	1,1	0,5	2,1	0	42,9	0	0
1.neg.1.B1.SSSS	0,5	0	0,5	0	0	0	0	2,6
27.neg.neg.B2.SRSS	3,3	1,1	NA	0	0	0	0	0
15.neg.neg.D.SRSS	0,9	2,3	NA	0	0	0	0	0
15.neg.neg.D.RRSR	0,5	1,1	NA	0	0	0	0	0
15.neg.neg.B2.SRSS	1,4	17,2	NA	0	0	0	0	0
15.neg.neg.B2.SRSR	1,4	9,2	NA	0	0	0	0	0
15.neg.neg.A.RRRR	0,5	2,3	NA	0	0	0	0	0
15.neg.1.D.SSSS	2,3	1,1	NA	0	0	0	0	0
15.neg.1.B2.SSSR	0,5	1,1	NA	0	0	0	0	0
15.neg.1.B2.SRSS	0,5	5,7	NA	0	0	0	0	0
15.neg.1.B2.SRSR	0,9	2,3	NA	0	0	0	0	0
15.neg.1.B1.SSSS	0,5	1,1	NA	0	0	0	0	0
15.neg.1.A.SRSR	3,3	1,1	NA	0	0	0	0	0
14.neg.neg.D.SRSS	0,5	1,1	NA	0	0	0	0	0
14.neg.neg.D.RSSR	1,4	1,1	NA	0	0	0	0	0
14.neg.neg.B2.SSSS	0,5	2,3	NA	0	0	0	0	0
14.neg.neg.B2.SRSS	0,5	1,1	NA	0	0	0	0	0
14.neg.1.D.RSSS	0,9	1,1	NA	0	0	0	0	0
1.neg.1.B2.SSSS	0,5	3,4	NA	0	0	0	0	0
Types from unknown sources	35,7	0	55,9	0	0	0	0	0
**Absolute number of isolates for various type categories**								
Matching + unknown types	**213**	**87**	**213**	**94**	**34**	**7**	**16**	**115**
Matching types	137	87	94	94	34	7	16	115
Types from unknown sources	76	0	119	0	0	0	0	0
Other types	0	125	0	89	19	22	65	49
All types	213	212	213	183	53	29	81	164

The heat map shows the proportions of isolates with certain types among the sum of isolates with matching and unknown types for each source and each group of cases. Classification is according to scheme Set 3 (resistance genes: “CTX-M.SHV.TEM.Phylogroup.ARP”), for Approach A (only animal sources) and Approach B (with nosocomial infections). NA = not applicable. *See description Table 3.*

Using ESBL genes, phylogenetic groups and antimicrobial resistance pattern (Set A3) most human cases could not be attributed to any of the animal sources. The proportion of attributed cases to the animal sources was 15.9% for pig, 3.3% for horse and 1.9% for dog, which was lower than in Set A2. In contrast, the cases attributed to cattle (19.2%) increased compared to the previous set. The attributed cases to chicken (4.2%) remained quite similar and the majority of the cases (55.6%) was attributed to an unknown source (see Set 3 of Approach A in Fig 1 and S1 and S2 Tables).

### Approaches considering nosocomial cases as a source

#### Set B1

For Approach B, 935 isolates were considered. Based on the information on the presence of the ESBL genes *bla*_CTX-M_, *bla*_SHV_ and *bla*_TEM_ (Set B1) 34 different subtypes were identified. Among these, 17 types (comprising 52 isolates) were categorized as other types and 2 types were unknown types (comprising 7 isolates). The remaining 876 isolates allocated to 15 matching types (Table 5) were used for running the model.

The introduction of nosocomial cases as new a “source” to the model redistributed a considerable number of human community cases to that new source (44.9%). Hence, the number of attributed cases to the other sources decreased. The model attributed less cases to dog (20.4%), cattle (9.3%), pig (8.8%), horse (8.8%), chicken (4.6%) and the unknown source (3.2%) compared to Set A1 (see Set 1 of Approach B in Fig 1 and S1 Table).

#### Set B2

Adding the information on the phylogenetic group (A, B1, B2 or D) of *E*. *coli* to the type definition (Set B2) resulted in 79 different subtypes with 8 unknown types (with 12 isolates) and 39 other types (with 68 isolates). The remaining 32 matching types (Table 4) were used to run the model.

Including the phylogenetic group into the subtype definition did not affect too much the number of attributed cases in the model. Compared to Set B1, there was a slight increase in the cases attributed to the nosocomial cases (46.9% versus 44.9%). In Set B2, the attributed cases to dog (17.8%), horse (5.6%) and pig (4.6%) also showed a slight decrease compared to Set B1. Surprisingly, a minor increase in the attributed cases was seen in cattle (12.7%) and chicken (6.6%) compared to Set B1. Only 5.6% of the human cases could not be explained by any source considered (see Set 2 of Approach B in Fig 1 and S1 Table).

#### Set B3

Adding the ARP to ESBL genes and phylogenetic group in the typing scheme (Set B3) lead to 238 subtypes, including 44 unknown types (with 76 isolates) and 139 other types (with 369 isolates). Overall, 55 matching types (Table 5) were used to run the model.

The introduction of the nosocomial source in Set B3 resulted in a significant reduction of the number of cases attributed to an unknown source (34.9%) compared to results in Set A3 (55.8%) (S1 and S2 Tables). When compared to Set B2, the proportion of unknown cases increased significantly (from 5.6% to 34.9%) and allocation to some sources decreased considerably, especially when it comes to dog (0.9%) and nosocomial (32.1%). Still, the nosocomial source accounted for almost as much cases as the sum of the animal sources (33.8%). Similar to Approach A, in Set B3, there were some increases in cases attributed to cattle (16.5%) and chicken (5.0%) compared to Set B2 (see Set 3 of Approach B in Fig 1 and S1 Table).

### Modifications of considered antibiotics

As expected, when only three antimicrobials are considered in our modified Approach B in defining the subtypes, the total number of subtypes decreased compared to Set B3 (with 238 subtypes). The Set B3 without chloramphenicol has 175 subtypes, without ciprofloxacin 188, without ertapenem 227 and without gentamicin 181. When it comes to matching types, the number of subtypes was the same between Set B3 and the Set B3 without chloramphenicol and the Set B3 without ciprofloxacin with 55 matching types each. The Set B3 without ertapenem had 57 matching types and the Set B3 without gentamicin had 49 matching types. Even though the number of matching types was similar, the composition of subtypes changed compared to the full Set B3.

#### Set B3 without chloramphenicol

**Fig 2:**
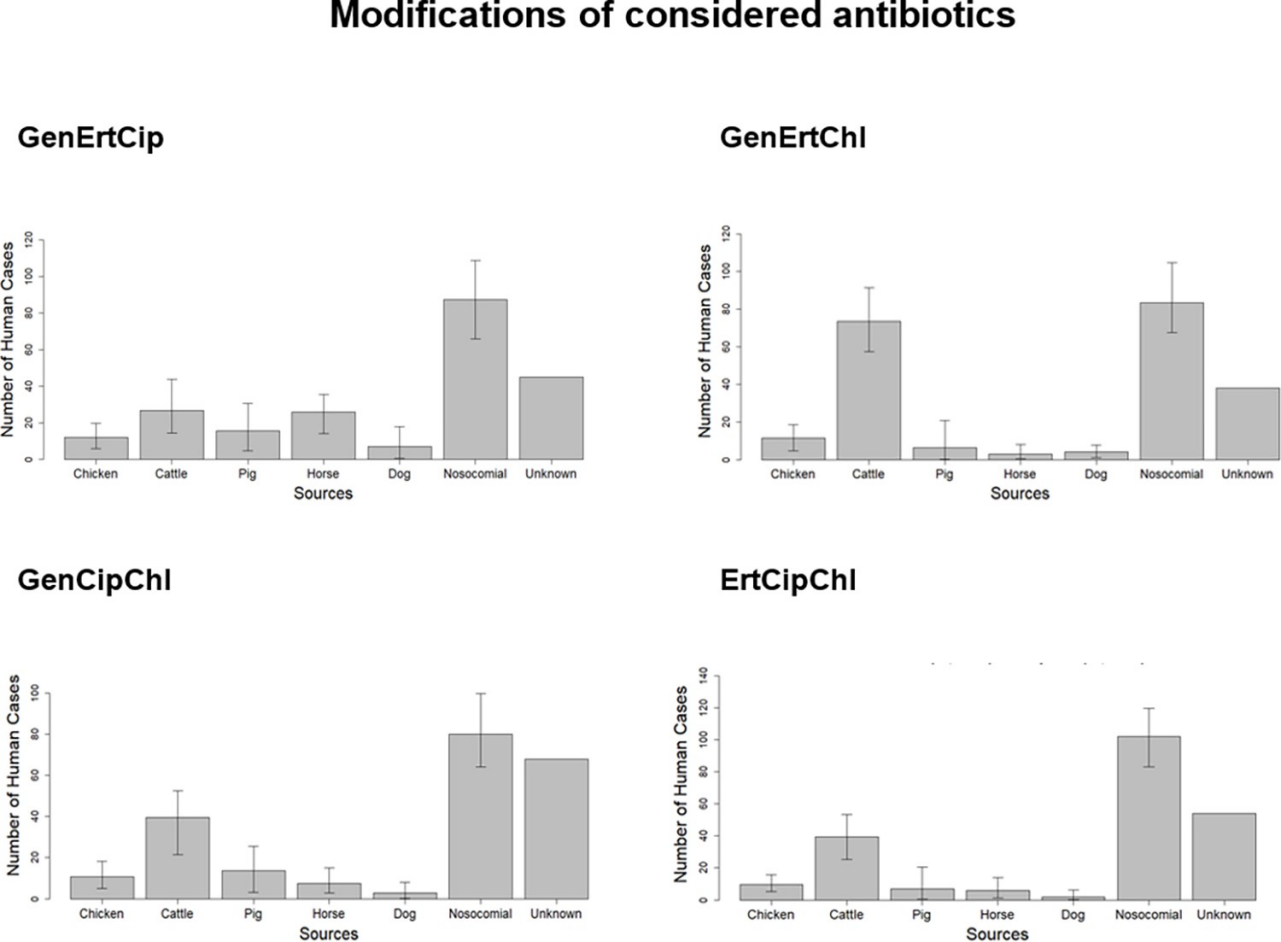
Source attribution modeling of ESBL-*E*. *coli* in human cases. Modified Approach B–each set considers the ESBL-type, phylogenetic group of *E*. *coli* and resistance pattern of three antimicrobials. Sources included are chicken, cattle, pig, horse, dog and hospitalized patients (nosocomial infections). GenErtCip, gentamicin, ertapenem, ciprofloxacin (without chloramphenicol); GenErtChl, gentamicin, ertapenem, chloramphenicol (without ciprofloxacin); GenCipChl, gentamicin, ciprofloxacin and chloramphenicol (without ertapenem); ErtCipChl, ertapenem, ciprofloxacin, chloramphenicol (without gentamicin).

Based on this dataset, nosocomial (38.7%), cattle and horse (12.0% each) were the sources that contributed the most to the human cases. Thus allocation of cases to nosocomial, horse and dogs increased whereas it decreased for cattle. Whereas still a considerable portion (20.7%) of cases weren´t attributed to any of the sources this was clearly lower than for the full dataset with 34.9% for the unknown source (see Fig 2 and S1 Table).

#### Set B3 without ciprofloxacin

Most community cases were attributed to nosocomial (38.8%) and to cattle (32.7%). The other sources were of minor relevance. Not considering ciprofloxacin in categorizing the isolates thus led to a shift towards cases attributed to cattle. The unknown cases (17.8%) were also considerably lower than in the full B3 dataset (34.9%) (see Fig 2 and S1 Table).

#### Set B3 without ertapenem

When not considering ertapenem, most of the matching types belonged to the phylogroup A (42.2%), followed by D (21.6%), B1 (20.3%) and B2 (16.0%). The changed categorization approach had an impact on the composition of the *bla*_CTX-M_ genes in the matching types. The proportion of *bla*_CTX-M-15_ carrying isolates increased slightly whereas it decreased for isolates carrying *bla*_CTX-M-1_. Several sources (dog, cattle, pig, nosocomial) contributed to this change. Most matching types were susceptible to all three antimicrobials (55.0%), 20.1% of isolates were resistant to at least one antimicrobial, 16.0% were resistant to two of the three substances and 8.9% of the isolates were resistant to all three substances.

The number of cases attributed to nosocomial (35.5%) was the highest, followed by cattle (18.4%), pig (6.9%), chicken (3.7%), horse (3.2%) and dog (0.9%). The cases not attributed to any of the sources (unknown) represented 31.3% of cases in the model (see Fig 2 and S1 Table). Compared to the full B3 model, there were no major changes in the allocated proportions to the individual sources.

#### Set B3 without gentamicin

When gentamicin was not considered in the grouping approach, most isolates belonged to the phylogroup A (43.1%), followed by B1 (23.3%), D (21.1%) and B2 (12.6%). Compared to the full Set B3, there was a shift towards isolates with phylogroup B1 in the matching types. Isolates from dogs, horses and nosocomial cases contributed to this. Compared to Set B3, the proportion of isolates carrying *bla*_CTX-M-1_ decreased. This was due to a higher frequency of isolates from horses, pigs and nosocomial cases. The majority of isolates in the matching types was susceptible to all three antimicrobials (51.8%). Isolates showing resistance to one antimicrobial were quite high (33.8%), resistance to two antimicrobials was present in 13.9% and full resistance was shown in 0.5% of the matching types.

Most of the cases were attributed to nosocomial subtypes (45.8%), followed by cattle (18.1%), chicken (5.1%), pig (3.2%), horse (1.9%) and dog (0.9%). A high portion of cases were attributed to a unknown source (25.0%) (see Fig 2 and S1 Table). Compared to the full B3 model, there was mainly a shift from unknown sources towards the nosocomial source.

## Discussion

This study focused on assessing the possible role of major food producing animals, companion animals and humans that have acquired a nosocomial infection as a source for colonization of humans with ESBL producing *E*. *coli*. The sources included in our study are considered at the first stage of the food chain, at the point of reservoir, since the samples collected in our study were taken directly from the animals or their direct environment and our approach in based on microbial subtyping [38]. Routes of transmission including slaughter, processing and retail were not included and the point of exposure in which the transmission happened (e.g. by direct contact or consumption) is not covered in our study [39].

An important finding of this study is that all considered animal sources (broiler, cattle, pig, dog and horse) and nosocomial isolates share most subtypes of ESBL-positive bacteria with the general population. This suggests that animal sources play an important role in the transmission of resistant bacteria. At the same time, we found out that human cases were more often attributed to nosocomial isolates than to any of the animal sources, implying a tendency for the human-to-human transmission and contribution in the transmission of ESBL-producing bacteria.

Another interesting point is that the estimated magnitude of this contribution largely depends on the information that is considered when defining subtypes. The more information is incorporated in defining the subtypes, the fewer is the number of community isolates that can be explained by the sources considered. Based on ESBL genes only, nearly 90% of the human cases can be explained by any of the sources included, which reflects the focus on the transferable element, which might emerge in several different bacterial backgrounds. When ESBL gene, phylogenetic group and resistance pattern are combined, this proportion decreases considerably, leaving around half of the cases without any matching source. This suggests that animals as a whole do not provide a straightforward route for the resistant bacteria found in humans. Rather it might be that smaller pockets in the animal population harbour the resistant bacteria found in humans. In this case it would be interesting to study where these pockets lie. Alternatively, it might be that complex process of bacterial evolution happens when bacteria move from animals to humans. Humans might serve as a melting pot for bacteria bringing together bacteria with different mobile elements from different animal populations.

### Role of animal sources in the model

In our study differences were observed in the contribution of the individual animal species as reservoir for community cases which was additionally depending on the information included in the subtype definition.

#### Pets (dogs and horses)

This observation is especially visible for dogs as a potential reservoir. When the ESBL type only (Set 1) or additionally the phylogenetic group (Set 2) are considered, dogs may contribute considerably to the human cases as a reservoir. This is linked with the fact that in our study sample, interestingly, isolates from dogs carried most frequently *bla*_CTX-M-15_, which was also the most common ESBL gene in our studied human populations. Contrary to our results, studies in the Netherlands [40, 41] identified *bla*_CTX-M-1_ as the most common *bla*_CTX-M_ gene amongst dogs. In line with our data, a study in Mexico [41, 42] identified mainly *bla*_CTX-M-15_ genes in healthy dogs and considered this ESBL-type to be emerging in animal species. Furthermore, other European studies highlighted the increase of *bla*_CTX-M-15_ in companion animals (including horses) in the last years [43, 44]. This increase in *bla*_CTX-M-15_ in pets follows the same line as in the human population, in which *bla*_CTX-M-15_ has been the dominating ESBL gene in the last years in Europe and in most parts of the world [45]. Moreover, when it comes to horses, in our study, *bla*_CTX-M-15_ was the second most common ESBL gene identified, preceded by *bla*_CTX-M-1_ and followed by *bla*_CTX-M-2_. Even though that horses contributed less than dogs to the human cases, the high proportion of *bla*_CTX-M-15_ in horses is similar with the results from dogs and differs from the livestock isolates in our study, which rarely carried *bla*_CTX-M-15_ genes. A study in the UK [46] described a change in the prevalence of genes from the *bla*_CTX-M_ group in horses over a ten-year period (from 2008 to 2017) in the same equine hospital. In 2008, none of the equine isolates carried the gene *bla*_CTX-M-15_, while in 2017 this ESBL gene was present in 60.8% of the analysed samples. Regarding *bla*_CTX-M-2_, this ESBL gene is also described in other studies [47–49] as an emerging ESBL gene in horses and mostly associated with clonal and nosocomial spread in equine clinics [47]. In our study, CTX-M-2-producing *E*. *coli* were present in both human populations but with a higher prevalence in the nosocomial isolates. Even though that the *bla*_CTX-M-2_ gene is not widely described in humans in Europe, other studies found it hospital facilities in Israel [50] and mostly in South America [51], where this gene from the CTX-M group is one of the most common amongst humans. Even though horses were not such a relevant source compared to dogs in our study, our findings shed some light on the similarities that exist between the human population and these two species in Germany and which ESBL genes may be most relevant when evaluating the potential role of pets in the transmission of ESBL-positive bacteria.

By adding the antimicrobial resistance pattern (Set 3) in our model, the similarity of isolates from humans and pets changes, especially in dogs which become almost irrelevant as a source to the human cases. This change is due to a considerable proportion of human cases being susceptible to all four considered antimicrobials while the subtype with the highest proportion in dogs was resistant to three antimicrobials. This may be explained since the dog isolates included in our study were clinical samples, while human cases were isolates from the healthy community. Thus, the high prevalence of multidrug-resistant *E*. *coli* in dogs may be due to previous or ongoing antibiotic therapy in the dogs included in our study [52].

#### Livestock (cattle, pigs and chicken)

When it comes to food producing animals, in our study, cattle and pigs may act as a reservoir for human exposure. The magnitude of this contribution depends on the range of possible sources included, as reflected in the difference between the outcome of Approach A, when only animal sources are considered and Approach B (discussed later). Most pig and cattle isolates carried *bla*_CTX-M-1_, which is the second most common ESBL gene in the human cases and similarly to the human cases, a high portion of cattle and pig isolates showed to be susceptible to the four tested antimicrobials. Nevertheless, when nosocomial isolates are considered in the model (Approach B), cattle and pig become less relevant as sources, highlighting the similarity between the nosocomial and the general community isolates in our study.

Chicken played in all approaches a minor role as reservoir for human cases. This can be explained by the predominance of ESBL genes, especially *bla*_SHV-12_ and *bla*_TEM-52_ in chicken isolates, but rarely in human cases. Our results are similar to recent studies by Mughini-Gras et al. and Dorado-Garcia et al. that showed that farm animals aren´t considered the most important sources when it comes to ESBL-producing *E*. *coli* and that human-to-human transmission should be considered as an important route instead [5, 40]. Yet, Mughini-Gras et al., Dorado-Garcia et al. and de Been et al., pointed out that farmers and farming communities may be more vulnerable to clonal transfer of ESBL genes due to having direct contact with livestock [5, 40, 53]. The same may apply for pets and pet owners.

### Transmission of ESBL genes between humans and animals

Our results show that the incorporation of ESBL genes (*bla*_CTX-M_, *bla*_SHV_ and *bla*_TEM_) and the phylogenetic group allows attribution of a broad number of human cases to animal sources. In contrast, the incorporation of phenotypic resistances made the source attribution less broad which was due to a growing dissimilarity between human and animal isolates in terms of phenotypic resistance patterns.

There is still lack of consensus about the direction of transmission of resistance determinants between pets and food animals to humans [54] and the mechanisms involved. In the last years, source attribution of resistance genes has been in the spotlight amongst the scientific community; nevertheless, most studies [54] have not been able to show evidence of the directionality of transmission of resistance genes between animals and humans. While some studies [55, 56] consider that direct contact with animals pose a risk to humans in the transmission of zoonotic bacteria such as ESBL-positive *E*.*coli*, other studies [57, 58] point out that the transmission pathway is most probable from humans to animals and not *vice-versa*. Better genomic data analysis (e.g. whole genome sequencing) combined with epidemiological details (e.g. spatiotemporal coherence of data in the populations; antibiotic usage in animals and humans) are needed to reconstruct the direction of transmission of resistance determinants between animals and people [54].

### Importance of human to human transmission

When incorporating human nosocomial cases as a potential source to get colonized with ESBL-producing *E*. *coli*, we observed that more cases were accounted to this source than by any animal source. The similarity of isolates in the community and nosocomial cases may reflect the close link between hospitalized patients and the general population, in that the general population might bring resistant bacteria into the hospital or that resistant bacteria are “bread” in the hospitals from which they come into the general population highlighting the relevance of human-to-human transmission [59]. Another possibility is that there is a common source for both populations.

In our study, we observed that CTX-M-15-producing *E*. *coli* in combination with phylogroup B2 was the most common subtype amongst nosocomial isolates and the second most common among the general population, while being very rare amongst animal sources. The epidemic lineage *E*. *coli* B2 025:H4-ST131, have been observed worldwide and is associated with the *bla*_CTX-M-15_ gene [45]. Although the ways of transmission and the mechanisms for the successful spread of *E*. *coli*-ST131 are still unclear, this lineage has shown to be associated with high levels of antimicrobial use and high prevalence in healthcare facilities [60]. Even though we didn´t consider sequence type (ST) information in our study, a previous study by Pietsch et al. which used the same human isolates as our study, identified *E*. *coli* 025:H4-ST131 in 32.3% of the nosocomial isolates [9]. The horizontal spread, specifically through IncF plasmids plays a major role for dissemination of *bla*_CTX-M-15_ in ST131 strains [61].

The similarity of isolates from the nosocomial and general population is characterized by subtypes carrying ESBL genes in both human populations that were absent in animals, such as *bla*_CTX-M-27_, *bla*_CTX-M-3_, *bla*_CTX-M-32_ and *bla*_CTX-M-55._ Even though all of these ESBL genes have been already identified in animal sources in other studies [62, 63], most of them were seldom reported and mostly outside of Europe [64–67]. An exception is observed in *bla*_CTX-M-55_, which was first identified as the second most common ESBL gene in food producing animals in China and later, also, as the second most common ESBL gene in community-associated infections in Chinese hospitals [68].

When it comes to *bla*_CTX-M-3_ in the human population, a report by Bevan et al. showed that, nowadays, CTX-M-3-producing *E*.*coli* is not common in Europe, even though its first identification happened in a Polish hospital [45, 69]. After its first appearance, this ESBL-type has been occasionally identified in humans in Asia [70, 71] and in Africa [72].

Regarding *bla*_CTX-M-27_, this ESBL gene was identified in humans in various regions of the world [73]. In Japan, a study by Matsumura et al. analysed ESBL-producing isolates in hospitals from 2001 to 2012 and identified CTX-M-27-producing *E*. *coli* as being associated with an ST131 clone epidemic in the country [74]. Environmental studies have also identified *bla*_CTX-M-27_ in Switzerland in aquatic samples [75], in well water in China [76] and in wastewater in Tunisia [77]. In our study, the *bla*_CTX-M-27_ gene was more common in the general community than in the nosocomial isolates and it was the most frequent ESBL gene amongst the unique human subtypes.

Lastly, *bla*_CTX-M-32_ was first isolated in a Spanish hospital [78]; nevertheless, there are few reports [79] that show the presence of this ESBL gene in humans in Europe. Other reports that identified *bla*_CTX-M-32_ were located in the American continent [80, 81] and in Asia [82]. When identified, this ESBL gene showed to be very rare amongst the isolates and was typically found in healthcare facilities.

### Variations due to subtype definition

By using different subtype definitions, we attempted to get a better understanding of the potential role of both, horizontal gene transfer and clonal spread of strains through different source attribution models.

On Set 1, when only the ESBL genes were considered, we assessed the transmission of genetic information related to the ESBL gene, which may be transferred through plasmids or other genetic elements. This showed that most of cases could be explained by the sources, while the number of cases not explainable (allocated to unknown sources) was the lowest. The similarity of ESBL genes between the sources and the cases in Set 1 may be related to the existence of epidemic plasmids that are successful in carrying resistant traits such as CTX-M-1 and CTX-M-15 in different hosts [83]. Epidemic plasmids are well adapted to different bacterial hosts, they have a tendency to acquire resistance genes and support the rapid dissemination of *Enterobacteriaceae* [84].

When including information present on the chromosome (e.g. phylogenetic group; Set 2) into the subtype definition, we noticed that this combination of ESBL genes with phylogenetic group lead to a lower number of attributed cases and an increase in cases with unknown sources. This reflects that the same ESBL genes are located in *E*. *coli* strains of different phylogenetic groups.

### Modifications in the considered antibiotics

Resistances to fluoroquinolones, including ciprofloxacin, have been described in *E*. *coli* of human origin especially associated with in urinary infections [85, 86]. A study by Arslan et al., found out that patients who received ciprofloxacin as a treatment were twice more susceptible to carrying ESBL-producing *E*. *coli* compared to the ones who did not [87]. When it comes to livestock, specifically cattle, resistance to fluoroquinolones, including ciprofloxacin have been also widely described in Germany [88, 89]. In veterinary medicine, fluoroquinolones (predominantly enrofloxacin) are used as a treatment for varied conditions, which show cross-resistance to ciprofloxacin [88]. Due to their importance for treating severe infections, fluoroquinolones are considered by the WHO [90] and OIE [91] as “Critically Important Antimicrobials” (CIA) for human and veterinary medicine, respectively. Hence, due to the concomitant clinical use of fluoroquinolones in humans and animals, other studies [92, 93], have considered that the surge in resistances in fluoroquinolones in humans in the past decades have emerged mostly because of the use and misuse of these substances in animals.

Interestingly, in our study, we observed that when ciprofloxacin was not considered in the subtype definition, there was a higher number of all susceptible isolates in the general population compared to the other sets. It was also observed; when ciprofloxacin was present in the modified sets, there were higher number of isolates with resistance linked to this substance in the human cases than resistance to any of the others antimicrobials. That means that significant amounts of human isolates showed ciprofloxacin resistance.

At the same time, when ciprofloxacin was removed from the considered group of antibiotics, we observed that the number of matching isolates between cattle and humans increased considerably which elevated the number of attributed cases to cattle. This reflects that ciprofloxacin resistance plays only a smaller role among the phenotypes in our cattle data.

Other studies in Germany [94, 95] from the RESET-consortium, have demonstrated that calves and dairy cattle have a higher chance to carry resistances than beef cattle. Also, another study in Germany [96] showed that, in general, clinical isolates in cattle have a higher prevalence of antimicrobial resistances than non-clinical isolates. When it comes specifically to ciprofloxacin, Tenhagen et al., observed that the highest levels of resistance were seen in calves with enteritis, followed by non-clinical veal calves and young cattle at slaughter, cows with mastitis, milk from bulk tanks and lastly in beef cattle [96].

In our study, we have excluded clinical isolates from cattle, which can partly explain the low levels of isolates with resistance to ciprofloxacin. Nevertheless, by allowing only samples from healthy animals in our model, we obtained a more realistic scenario since the probability of sick animals getting in contact and/or being consumed by the general population is rather scarce.

We haven´t divided the cattle population into dairy and beef, leaving us with no conclusions about the role of these two production types.

Furthermore, the low number of ciprofloxacin-resistant cattle isolates in our study may be due to the composition of our data set or a positive consequence of the nationwide measures in Germany to minimize the use of antimicrobials in animals [97]. Between the years 2011 and 2017 a fell by 57% of the amount of antimicrobials supplied to veterinarians and a decrease of resistance to ciprofloxacin was registered in *E*. *coli* from calves [97]. Also, other countries in Europe, such as Denmark, have already restricted the use of fluoroquinolones in food animals and managed to reduce significantly the use of these substances in livestock in the past years [98]. Decreases in fluoroquinolones’ consumption in the primary healthcare sector has been also registered in Denmark for the last years and their use is recommended only in specific situations [99]. Nevertheless, resistances against fluoroquinolones are still observed in Denmark but mostly related to travelers that come from abroad; resistances in food production animals are also existent in Denmark but in lower amounts when compared to humans [99]. In Australia, fluoroquinolones are forbidden to be administered to animals [98] and levels of resistances in the community are lower than in other countries but still present [100].

Most studies [101] concerning the origin of ciprofloxacin-resistant *E*. *coli* in the general population are related to community and hospital-acquired urinary tract infections (UTI). This supports our results, given that most ciprofloxacin-resistant isolates in the community were attributed to nosocomial isolates. Other European studies reported that the strongest predictors for acquiring ciprofloxacin-resistant *E*. *coli* in patients suffering with urinary tract infections were previous hospitalization in the last 3–6 months, the age of 51–65 and previous antibiotic treatment during the last 12 months [102, 103]. Another study in the Netherlands found that community-acquired urinary tract infections due to ciprofloxacin-resistant *E*. *coli* were associated with a high intake of pork and chicken, but not with beef [104].

Herein, we have observed that isolates from human cases and cattle showed to be phenotypically different when it comes to resistance to ciprofloxacin. For that reason, we conclude that cattle is probably an overestimated source to the human population when ciprofloxacin is not considered in the subtype definition. More studies that evaluate and compare the level of resistances to fluoroquinolones between the cattle and human populations and their potential catalysts (e.g. farm/slaughterhouse management and hygiene, antimicrobial use and administration method) and transmission routes (e.g. direct contact, meat consumption, cross-contamination and environmental sources) are necessary.

### Limitations

This study reflects the benefits of harmonized laboratory protocols for typing of isolates from different sources and across disciplines. The results achieved with the model can be just as good as the information included.

The dataset available has its limitations as regards representability of community cases for Germany, being Bavaria the only region covered, and the total number of isolates with complete information. Some animal species had more representation than others (e.g. dogs had a very low number of isolates compared to the other sources). Certainly, there is the risk to miss rare subtypes in a population due to the limited number of isolates included.

Furthermore, miss-classifications may have happened as resistance genes may have gone lost or phenotypic expression of resistance may have been triggered differently. Also, one laboratory analysed the human isolates while several others analysed the animal isolates; this may have led to different methods of antimicrobial susceptibility testing, even though the resistance breakpoints applied for interpretation of the data were the same.

Moreover, other sources were not included in this study, such as insects, that are considered a good indicator for environmental contamination [105], turkey, seafood, wildlife or vegetables, which may explain some of the unknown subtypes. As regards humans, there might be additional populations not included in the study e.g. travellers and people working in close contact with animals.

Our findings raised many questions, which cannot be answered by the present data. In further studies, it has to be investigated whether the prevalence of subtypes uniquely identified in humans continues to be the same, and if subtypes often found in animals continue to be rare in humans and *vice versa*. Furthermore, it needs to be addressed how important other sources are, such as vegetables, the environment and other animals, which could not be considered in this study. It remains unclear if and how often ESBL-producing bacteria are transmitted between animals and humans and how much they are subject to genetic modifications. There is also a lack of knowledge about the quantitative horizontal and vertical transmission rates of resistant bacteria and its mobile elements and the probability of a colonisation or an infection with such an organism. The existence and prevalence of epidemic lineages, such as *E*. *coli* 025:H4-ST131 amongst animals and humans also needs further investigation.

We could not make any inferences about the direction of transmission nor the complexity of the transmission cycles happening between the sources and humans, since we didn’t have additional epidemiological data and also not time series were available.

## Conclusion

In our work, we showed the potential impact of some animal sources on the transmission of ESBL *E*. *coli* to humans. We additionally explored a possible path for human-to-human transmission of ESBL-producing bacteria. By using different subtype definitions, we attempted to get a better understanding of the role of both, horizontal gene transfer and clonal spread of strains through source attribution models. At the same time different subtypes definitions lead to varying attribution results which highlights the complexity of the transmission pathways of ESBL-positive bacteria. This complexity calls for a One Health approach to study further the development and transmission of antimicrobial resistance.

## Supporting information

S1 Table Number of human cases attributed to each source by the source attribution model and their proportions (%). **Approach A** (Set A1, A2, A3) considers only animals (chicken, cattle, pig, horse and dog) as potential sources for the human cases; **Approach B** (Set B1, B2, B3) considers all the sources from Approach A and isolates from nosocomial infections. **Set 1**, considers only ESBL-types; **Set 2**, considers ESBL-types and phylogenetic group of *E*.*coli*; **Set 3**, considers ESBL-types, phylogenetic group of *E*.*coli* and resistance pattern of four antimicrobials. **Set GenErtCip** includes gentamicin, ertapenem, ciprofloxacin (without chloramphenicol); **Set GenErtChl,** includes gentamicin, ertapenem, chloramphenicol (without ciprofloxacin); **Set GenCipChl**, includes gentamicin, ciprofloxacin and chloramphenicol (without ertapenem); **Set ErtCipChl**, includes ertapenem, ciprofloxacin, chloramphenicol (without gentamicin). The isolates attributed to the unknown source represent the human cases which could not be attributed to any of the sources in the study.
(DOCX)

S2 Table Unknown types and respective number of cases that could not be attributed to any of the sources. The nosocomial isolates represent the number of isolates present in this source on Set B3. The grey shaded subtypes represent the isolates that on Set A3 were unknown but then on Set B3 were attributed to the nosocomial source. These subtypes shifted from unknown to matching types when the nosocomial was introduced as a source.
(DOCX)

S1 File Excel workbook with unique subtypes in each set and respective number of isolates per source and human cases. The data about subtypes present in both human cases and sources in this excel workbook were used in the source attribution model.
(XLSX)
