# Supplementary material for: Comparison of approaches for source attribution of ESBL-producing Escherichia coli in Germany
Source: PLoS One. 2022 Jul 15;17(7):e0271317. doi: 10.1371/journal.pone.0271317 (PMC9286285; doi:10.1371/journal.pone.0271317)
Supplement: S1 Table — Approach A (Set A1, A2, A3) considers only animals (chicken, cattle, pig, horse and dog) as potential sources for the human cases; Approach B (Set B1, B2, B3) considers all the sources from Approach A and isolates from nosocomial infections. Set 1, considers only ESBL-types; Set 2, considers ESBL-types and phylogenetic group of E.coli; Set 3, considers ESBL-types, phylogenetic group of E.coli and resistance pattern of four antimicrobials. Set GenErtCip includes gentamicin, ertapenem, ciprofloxacin (without chloramphenicol); Set GenErtChl, includes gentamicin, ertapenem, chloramphenicol (without ciprofloxacin); Set GenCipChl, includes gentamicin, ciprofloxacin and chloramphenicol (without ertapenem); Set ErtCipChl, includes ertapenem, ciprofloxacin, chloramphenicol (without gentamicin). The isolates attributed to the unknown source represent the human cases which could not be attributed to any of the sources in the study. (DOCX) [file pone.0271317.s001.docx]

**S1 Table**

|  |  | **Sources** | | | | | | | |  |
| --- | --- | --- | --- | --- | --- | --- | --- | --- | --- | --- |
|  |  | Chicken | | Cattle | Pig | Horse | Dog | Nosocomial | Unknown | Cases* |
| **Sets** | **A1** | | 11 (5.1%) | 52 (24.3%) | 28 (13.1%) | 26 (12.1%) | 75 (35.0%) | - | 22 (10.3%) | 214 |
|  | **A2** | | 8 (3.8%) | 24 (11.3%) | 61 (28.6%) | 24 (11.3%) | 64 (30.0%) | - | 32 (15.0%) | 213 |
|  | **A3** | | 9 (4.2%) | 41 (19.2%) | 34 (15.9%) | 7  (3.3%) | 4  (1.9%) | - | 119 (55.6%) | 214 |
|  | **B1** | | 10 (4.6%) | 20 (9.3%) | 19 (8.8%) | 19 (8.8%) | 44 (20.4%) | 97  (44.9%) | 7  (3.2%) | 216 |
|  | **B2** | | 14 (6.6%) | 27 (12.7%) | 10 (4.7%) | 12 (5.6%) | 38 (17.8%) | 100 (46.9%) | 12  (5.6%) | 213 |
|  | **B3** | | 11 (5.0%) | 36 (16.5%) | 15 (6.9%) | 8  (3.7%) | 2  (0.9%) | 70  (32.1%) | 76 (34.9%) | 218 |
|  | **GenErtCip** | | 11 | 26 | 15 | 26 | 10 | 84 | 45 | 217 |
|  |  |  | (5.1%) | (12.0%) | (6.9%) | (12.0%) | (4.6%) | (38.7%) | (20.7%) |  |
|  | **GenErtChl** | | 12 | 70 | 5 | 2 | 4 | 83 | 38 | 214 |
|  |  |  | (5.6%) | (32.7%) | (2.3%) | (0.9%) | (1.9%) | (38.8%) | (17.8%) |  |
|  | **GenCipChl** | | 8 | 40 | 15 | 7 | 2 | 77 | 68 | 217 |
|  |  |  | (3.7%) | (18.4%) | (6.9%) | (3.2%) | (0.9%) | (35.5%) | (31.3%) |  |
|  | **ErtCipChl** | | 11 | 39 | 7 | 4 | 2 | 99 | 54 | 216 |
|  |  |  | (5.1%) | (18.1%) | (3.2%) | (1.9%) | (0.9%) | (45.8%) | (25.0%) |  |

** The total number of cases in each set varies since the model is based on a stochastic simulation (Markov Chain Monte Carlo).*
