# Supplementary material for: Comparison of approaches for source attribution of ESBL-producing Escherichia coli in Germany
Source: PLoS One. 2022 Jul 15;17(7):e0271317. doi: 10.1371/journal.pone.0271317 (PMC9286285; doi:10.1371/journal.pone.0271317)
Supplement: S2 Table — The nosocomial isolates represent the number of isolates present in this source on Set B3. The grey shaded subtypes represent the isolates that on Set A3 were unknown but then on Set B3 were attributed to the nosocomial source. These subtypes shifted from unknown to matching types when the nosocomial was introduced as a source. (DOCX) [file pone.0271317.s002.docx]

**S2 Table**

| **Subtype** | **Cases** | **Nosocomial Isolates** |
| --- | --- | --- |
| 15.neg.1.A.SRSR | 7 | 1 |
| 27.neg.neg.B2.SRSS | 7 | 1 |
| 15.neg.1.D.SSSS | 5 | 1 |
| 15.neg.neg.B2.SRSS | 3 | 15 |
| 15.neg.neg.B2.SRSR | 3 | 8 |
| 14.neg.neg.D.RSSR | 3 | 1 |
| 15.neg.1.B2.SRSR | 2 | 2 |
| 15.neg.neg.D.SRSS | 2 | 2 |
| 14.neg.1.D.RSSS | 2 | 1 |
| 15.neg.1.B2.SRSS | 1 | 5 |
| 1.neg.1.B2.SSSS | 1 | 3 |
| 14.neg.neg.B2.SSSS | 1 | 2 |
| 15.neg.neg.A.RRRR | 1 | 2 |
| 14.neg.neg.B2.SRSS | 1 | 1 |
| 14.neg.neg.D.SRSS | 1 | 1 |
| 15.neg.1.B1.SSSS | 1 | 1 |
| 15.neg.1.B2.SSSR | 1 | 1 |
| 15.neg.neg.D.RRSR | 1 | 1 |
| 15.neg.1.D.SSSR | 7 | 0 |
| 15.neg.1.A.SRSS | 6 | 0 |
| 15.neg.1.A.SSSS | 6 | 0 |
| 14.neg.1.D.SSSS | 5 | 0 |
| 15.neg.1.D.SRSR | 4 | 0 |
| 15.neg.1.A.RSSR | 3 | 0 |
| 15.neg.1.D.SRSS | 3 | 0 |
| 14.neg.1.A.SSSS | 2 | 0 |
| 14.neg.neg.D.RSSS | 2 | 0 |
| 15.neg.1.B2.SSSS | 2 | 0 |
| 3.neg.1.D.SRSS | 2 | 0 |
| 3.neg.1.D.SSSS | 2 | 0 |
| 1.neg.1.B2.SRSS | 1 | 0 |
| 1.neg.1.D.RSRS | 1 | 0 |
| 1.neg.neg.D.RSRS | 1 | 0 |
| 14.neg.1.D.RSRS | 1 | 0 |
| 14.neg.neg.D.RRSR | 1 | 0 |
| 14.neg.neg.D.SSSR | 1 | 0 |
| 15.2.neg.A.SSSS | 1 | 0 |
| 15.neg.1.A.SRRR | 1 | 0 |
| 15.neg.1.A.SSRS | 1 | 0 |
| 15.neg.1.A.SSSR | 1 | 0 |
| 15.neg.1.B2.RSSS | 1 | 0 |
| 15.neg.1.D.RSSS | 1 | 0 |
| 15.neg.neg.A.SRRR | 1 | 0 |
| 15.neg.neg.A.SSRS | 1 | 0 |
| 15.neg.neg.A.SSSR | 1 | 0 |
| 15.neg.neg.D.SSSR | 1 | 0 |
| 2.neg.neg.A.SSSR | 1 | 0 |
| 27.neg.neg.B2.SSSS | 1 | 0 |
| 3.neg.1.A.SRSS | 1 | 0 |
| 3.neg.1.D.SSSR | 1 | 0 |
| 3.neg.neg.A.SSSS | 1 | 0 |
| 3.neg.neg.B2.SRSR | 1 | 0 |
| 3.neg.neg.D.RSSR | 1 | 0 |
| 3.neg.neg.D.RSSS | 1 | 0 |
| 3.neg.neg.D.SSSR | 1 | 0 |
| 32.neg.neg.A.SRSS | 1 | 0 |
| 55.neg.1.B2.SSSS | 1 | 0 |
| 9.neg.1.D.SSSS | 1 | 0 |
| neg.12.1.A.SRSS | 1 | 0 |
| neg.12.neg.B1.RSSS | 1 | 0 |
| neg.neg.52.A.SRSS | 1 | 0 |
| neg.neg.52.D.SSSR | 1 | 0 |
